# Uncovering pathophysiological changes in frontotemporal dementia using serum lipids

**DOI:** 10.1038/s41598-020-60457-w

**Published:** 2020-02-27

**Authors:** Katherine Phan, Ying He, Russell Pickford, Surabhi Bhatia, Jared S. Katzeff, John R. Hodges, Olivier Piguet, Glenda M. Halliday, Woojin Scott Kim

**Affiliations:** 1 The University of Sydney, Brain and Mind Centre & Central Clinical School, Sydney, NSW Australia; 2 Bioanalytical Mass Spectrometry Facility, University of New South Wales, Sydney, NSW Australia; 3 ARC Centre of Excellence in Cognition and its Disorders, Sydney, NSW Australia; 4 The University of Sydney, Brain and Mind Centre & School of Psychology, Sydney, NSW Australia; 5 Neuroscience Research Australia, Sydney, NSW Australia; 6 School of Medical Sciences, University of New South Wales, Sydney, NSW Australia

**Keywords:** Lipidomics, Development of the nervous system

## Abstract

Blood serum is enriched in lipids and has provided a platform to understand the pathogenesis of a number of human diseases with improved diagnosis and development of biomarkers. Understanding lipid changes in neurodegenerative diseases is particularly important because of the fact that lipids make up >50% of brain tissues. Frontotemporal dementia (FTD) is a common cause of early onset dementia, characterized by brain atrophy in the frontal and temporal regions, concomitant loss of lipids and dyslipidemia. However, little is known about the link between dyslipidemia and FTD pathophysiology. Here, we utilized an innovative approach – lipidomics based on mass spectrometry – to investigate three key aspects of FTD pathophysiology – mitochondrial dysfunction, inflammation, and oxidative stress. We analyzed the lipids that are intrinsically linked to neurodegeneration in serum collected from FTD patients and controls. We found that cardiolipin, acylcarnitine, lysophosphatidylcholine, platelet-activating factor, o-acyl-ω-hydroxy fatty acid and acrolein were specifically altered in FTD with strong correlation between the lipids, signifying pathophysiological changes in FTD. The lipid changes were verified by measurement of the common disease markers (e.g. ATP, cytokine, calcium) using conventional assays. When put together, these results support the use of lipidomics technology to detect pathophysiological changes in FTD.

## Introduction

Lipidomics is a systems-level analysis and characterization of lipids^1,2^. Like other “omics” analyses, such as transcriptomics and proteomics, lipidomics is a global profiling of lipid species present in cells, tissues or body fluids^3^. Lipidomics allows detection and quantification of thousands of different lipid species of a broad range of lipid classes^4^. It has facilitated a greater understanding of the pathophysiology of a range of human diseases, particularly cardiovascular disease and diabetes. It has also provided invaluable data to identify risk factors and develop biomarkers for disease identification and classification. Recent advances in lipidomics technology based on mass spectrometry have significantly improved the detection of a vast array of lipids present in blood serum. Importantly, it has allowed detection of small, yet significant, differences in lipid levels that are intrinsically linked to disease processes. Application of lipidomics in the study of neurodegenerative diseases is particularly imperative because of the fact that lipids make up 39.6% of the brain grey matter and 64.6% of the white matter^5^.

Frontotemporal dementia (FTD) is the second most common form of younger-onset dementia after Alzheimer’s disease (AD), of which the main clinical syndrome is the behavioral variant (bvFTD)^6^. Clinically, bvFTD is characterized by progressive changes in behavior and personality, loss of empathy, apathy, as well as variable cognitive deficits that include executive function, language and, in a subset episodic memory. Pathologically, FTD is characterized by brain atrophy in the frontal and temporal regions, and concomitant loss of lipids^7–10^. Although some of the clinical features of FTD overlap with those found in AD, the underlying brain cellular pathologies are distinctive in the two diseases. In FTD, aggregates of microtubule associated protein tau (MAPT), tar DNA-binding protein-43 (TDP43) or fused in sarcoma (FUS) are present in the brain; whereas in AD, aggregates of amyloid-β peptides or neurofibrillary tangles (composed of tau) are present in the brain.

Blood fluids of FTD patients have been analyzed using conventional lipid assays based on enzymes, and these have shown that triglyceride (TG) levels are increased in FTD compared to controls^11^. Global lipid analysis has also shown that TG levels are increased in FTD compared to controls, along with changes in other lipids^12^. Furthermore, correlation studies have shown that TG levels are positively correlated with body mass index (BMI), whereas HDL cholesterol levels are negatively correlated with BMI^11^. Both TG and HDL cholesterol levels are also correlated to eating behavior (fat intake) and measures of cognition and disease duration^13^. Although these findings suggest manifestation of lipid dysregulation, the exact relationship between lipid dysregulation and FTD pathophysiology is largely unknown.

In the current study, we undertook lipidomics analysis of FTD serum to investigate three key aspects of FTD pathophysiology relevant to neurodegeneration: mitochondrial dysfunction, inflammation, and oxidative stress. We focused on specific lipids that are intrinsically involved in each of these three pathological processes. In the mitochondrial dysfunction study, we analyzed cardiolipin and acylcarnitine, both of which are involved in mitochondrial energy production^14,15^. In the inflammation study, we analyzed two pro-inflammatory lipids lysophosphatidylcholine and platelet-activating factor; both play important roles in innate immunity in the host defense cells^16,17^. Finally, in the oxidative stress study, we examined the three major lipid aldehydes, acrolein, malondialdehyde and 4-hydroxynonenal, all of which are known to cause oxidative damage to cells. The first aim of our study was to detect pathophysiological changes in FTD using serum lipids. The second aim was to uncover lipid species and lipid synthesis pathways that could be exploited to develop biomarkers for FTD.

## Materials and Methods

### Chemicals and materials

Lipids were extracted using chloroform, methanol and isopropanol (Sigma Aldrich, St. Louis, MO, USA) and ultrapure water (Millipore). All solvents used were HPLC grade or higher. Glass pipettes and tubes were used wherever possible and the use of plasticware was minimized during lipid extraction to avoid contamination of samples. Glass tubes and glass transfer pipettes were purchased from Sigma and vWR. Lipid internal standards (ISTDs) were purchased from Avanti Polar Lipids Inc. (Alabaster, AL, USA). These include phosphatidylcholine (19:0), sphingomyelin (12:0), phosphatidylethanolamine (17:0), phosphatidylglycerol (17:0), phosphatidylserine (17:0), phosphatidic acid (17:0), ceramide (d18:1, 12:0), diglyceride (1,3 18:0 d5), cholesteryl ester (19:0), monoglyceride (17:0), triglyceride mix d5 (Avanti Code LM-6000), diglyceride mix d5 (Avanti Code LM-6001), phosphatidylinositol (17:0 14:1), C12 GluCer, C12 sulfatide, C17 ceramide, C17 sphingosine, C17 S1P, C12 C1P, D3 C20 fatty acid, and C12 LacCer. Lipid internal standards were prepared as a mixture at 10 pmol/µl in methyl-tert butyl ether and methanol (MTBE:methanol, 1:1 v/v).

### Patient blood serum

Individuals diagnosed with bvFTD (N = 40) and healthy controls (N = 22) were recruited at Neuroscience Research Australia in Sydney from FRONTIER, the frontotemporal dementia clinical research group, and from a panel of healthy study volunteers^11^ with no neurological (i.e. no evidence of cognitive impairment) or psychiatric disorders. The study was approved by the University of New South Wales human ethics committee (approval number: HC12573). All methods were carried out in accordance with the relevant guidelines and regulations. Blood samples were obtained following written informed consent from the participant and/or primary carer. All patients underwent a neurological examination, a comprehensive cognitive assessment and structural brain MRI, and met current consensus diagnostic criteria for bvFTD^18^, as previously described^11^. The mean age at assessment was 65 ± 8 years for bvFTD and 71 ± 5 years for controls. Blood samples (9 mL) were collected in tubes (BD Vacutainer SST II Advance Tube #367958), and serum prepared by centrifugation at 3,500 rpm for 10 min at 4 °C, which was then aliquoted and stored at −80 °C until use.

### Human brain tissues

Frozen post-mortem brain tissue samples were obtained from Sydney Brain Bank and NSW Brain Tissue Resource Centre following appropriate ethical approvals (University of New South Wales Human Research Ethics approval number: HC15789). Frozen samples from the superior frontal cortex from 10 FTD cases, 10 AD cases and 11 controls without neurological, psychiatric or neuropathological diagnoses^19,20^ were used in this study. The mean age of the three groups were 72.9 ± 13.0, 73.7 ± 7.5 and 79.5 ± 12.1 years, respectively.

### Lipid extraction

Serum lipid extraction was based on the Bligh and Dyer method^21^. Briefly, serum samples were thawed on ice and 80 µl aliquots were transferred into glass tubes. Methanol (600 µl), chloroform (1,000 µl) and ultrapure water (500 µl) were sequentially added with vortexing between each addition. Samples were then centrifuged at 3,000 rpm for 10 min at room temperature. The lower solvent phase was collected and transferred to a new glass tube using a glass Pasteur pipette. Chloroform (600 µl) was added to the upper phase, vortexed and centrifuged at 3,000 rpm for 10 min. The lower phase was collected and transferred into the same glass tube and dried under nitrogen gas. Dried lipid samples were reconstituted in 100 µl of isopropanol/methanol (1:1) and stored at −80 °C in glass LC-MS vials.

### Lipidomics mass spectrometry

Lipid extracts (10 μl) were analyzed using a Q-Exactive Plus Mass Spectrometer coupled to a U3000 UPLC system (ThermoFisher Scientific). Chromatography was performed at 60 °C on a Waters CSH C18 UHPLC column 2.1 ×100 mm, 1.8 μM with VanGuard guard column. Solvent A was 6:4 acetonitrile:water and Solvent B was 1:9 acetonitrile:isopropanol, both with 10 mM ammonium formate and 0.1% formic acid. Lipids were chromatographed according to the method of Castro-Perez *et al*.^22^. Briefly, a 30 min gradient running from 30 to 100% of solvent B was performed, eluting lipids in order of hydrophobicity. Column eluate was directed into the electrospray ionization source of the mass spectrometer where a HESI probe was employed. Source parameters were broadly optimized on a range of lipid standards prior to the analysis. The mass spectrometer was run in data dependent acquisition mode. A survey scan over the mass range 200–1,200 at resolution 70 K was followed by 10 data dependent MS/MS scans on the most intense ions in the survey at 15 K resolution. Dynamic exclusion was used to improve the number of ions targeted. Cycle time was approximately 1 sec. Samples were run in both positive and negative polarities. The samples were run in a random order (generated using Microsoft Excel). This is important to avoid batch effects/changing instrument performance effects. Data were analyzed in LipidSearch software 4.1.16. Data were searched against the standard Lipidsearch database with all common mammalian lipid classes included. The search results were then grouped according to sample type and aligned for differential analysis. Aligned data (containing lipid identity, retention time, peak area etc.) were exported to Excel software. Relative abundance of lipids was obtained from peak areas normalized to internal standards.

### Thin layer chromatography

Firstly, serum lipid concentrations were determined using the Sulfo-Phospho-Vanillin (SPV) method as previously described^23^. Briefly, 10 µl of lipid extracts were loaded into a microplate and the solvent removed by evaporation at 90 °C. Concentrated sulphuric acid (100 µl) was added and incubated for 20 min at 90 °C. The plate was rapidly cooled to room temperature and background absorbance measured at 540 nm. 50 µl of vanillin-phosphoric reagent (20% (w/v) vanillin in 17% (v/v) phosphoric acid) was added, the color developed for 10 min and absorbance measured at 540 nm. Total lipid was calculated based on a standard curve generated using fish oil (Blackmores). The serum lipids were then separated using thin layer chromatography (TLC) as previously described^24^. Briefly, volumes of extracted lipids equivalent to 20 µg were spotted onto TLC plates (Silca gel 60, Merck). The plates were developed firstly in chloroform/methanol/water (40:10:1) to 2 cm, then in chloroform/methanol/water (40:10:1) to 5 cm, followed by chloroform/methanol/acetic acid (47:2:0.5) to 8.5 cm and lastly n-hexane/diethyl ether/acetic acid (65:35:1) to the top of the plates. Lipids were visualized by misting the plate with 5% (w/v) CuSO_4_ in 15% (w/v) H_3_PO_4_ and heating for 10 min at 180 °C. Band intensities were determined using BioRad Image Lab.

### ATP assay

Fluorometric ATP assay was carried out following the manufacturer’s protocol (Abcam, cat. # ab83355). Briefly, 50 µl of samples and standards were added to 96-well plates containing the ATP reaction mix and incubated at room temperature in the dark for 30 min. The plates were read using CLARIOstar microplate reader (BMG Labtech) at Ex/Em = 535/587 nm.

### Inflammatory marker assays

IL-6 was measured using the magnetic human cytokine ELISA assay (Bio-Rad) following the manufacturer’s instructions. Plates containing the serum samples and standards were analyzed using Magpix plate reader (Luminex), and the concentration of IL-6 was determined using the Xponent software package (Luminex). C3 was measured by western blotting (see below). Calcium assay was carried out following the manufacturer’s protocol (Abcam, ab102505). Briefly, 50 µl of samples and standards were added to 96-well plates. Then, 90 µl of the chromogenic reagent was added to each well, followed by 60 µl of calcium assay buffer. The plates were incubated at room temperature for 10 min in the dark and read in a microplate reader at 575 nm.

### Protein extraction

Tris-buffered saline (TBS)-soluble proteins were extracted from 100 mg of brain tissues as previously described^25^. Briefly, tissue samples were homogenized in ten volumes of TBS homogenization buffer (20 mM Tris, 150 mM NaCl, pH 7.4, 5 mM EDTA, 0.02% sodium azide) containing protease inhibitor cocktail (Roche) using Qiagen TissueLyser (3 × 30 sec, 30 Hz cycles), followed by centrifugation at 100,000 × g for 1 h at 4 °C. The supernatant was collected and transferred into new tubes. Protein concentration was measured using a bicinchoninic acid assay (Pierce BCA Protein Assay Kit) following the manufacturer’s instructions.

### Western blotting

Serum (equal volumes) or protein lysates (10 µg) were heated with sample buffer (3.2% SDS, 32% glycerol, 0.16% bromophenol blue, 100 mM Tris-HCl, pH 6.8, 8% 2-mercaptoethanol). They were then electrophoresed on Criterion Stain-free 4–20% SDS-PAGE gels (Bio-Rad) and transferred onto nitrocellulose membranes at 100 volts for 30 min. The membranes were blocked with TBS containing 5% nonfat dry milk and probed with anti-C3 antibody (Santa Cruz, sc-28294, 1:1,000) or anti-acrolein antibody (NOVUS, NB200–556, 1:1,000) overnight at 4 °C. They were then washed three times in TBS containing 0.1% Tween 20 and incubated with horseradish peroxidase-conjugated secondary antibody for 2 h at room temperature. Signals were detected using enhanced chemiluminescence and Gel Doc System (Bio-Rad). The blots were stripped and probed for housekeeper proteins transferrin (serum) or β-actin (tissue lysate). The signal intensity was quantified using Image Lab (Bio-Rad) and NIH ImageJ software (v1.45 s).

### Malondialdehyde assay

Malondialdehyde (MDA) was measured using Lipid Peroxidation MDA Assay Kit (Abcam, cat. # ab118970) following the manufacturer’s protocol. Briefly, samples were prepared by addition of 500 µL of 42 mM H_2_SO_4_ and 125 µL of phosphotungstic acid solution to 50 µL of serum samples. After incubation and centrifugation, the pellet was dissolved in 200 µL of ddH_2_O by sonication. TBA solution was then added into each vial containing samples or standards and incubated at 95 °C for 1 h. Fluorescence at excitation 532 nm/emission 553 nm was read using CLARIOstar plate reader (BMG Labtech).

### 4-Hydroxynonenal assay

4-Hydroxynonenal (HNE) was measured using OxiSelectTM HNE Adduct Competitive ELISA Kit (Cell Biolabs, Inc., San Diego, CA) following the manufacturer’s protocol. Briefly, 50 µL of serum was added to HNE Conjugate pre-coated wells followed by addition of 50 µL of anti-HNE antibody. After 1-h incubation at room temperature and washing, 100 µL of secondary antibody-HRP conjugate was added to each well and incubated for 1 h at room temperature. The plate was washed three times. 100 µL of substrate solution followed by stop solution were added into each well. Absorbance at 450 nm was read on POLARstar Omega plate reader (BMG Labtech).

### Statistical analysis

Statistical analyses were performed using SPSS Statistics software (IBM, Chicago, Illinois). For comparisons between FTD and control groups, either univariate analysis (general linear model) or Student’s *t*-test was used and statistical significance set at *p* < 0.05. When univariate analysis was performed, age and gender were included as covariates. Pearson’s correlations were used to determine if changes in lipid levels were associated with each other with statistical significance set at *p* < 0.05. Graphs were generated using GraphPad Prism 7.

## Results

### Validation of lipid analysis of FTD serum

**Figure 1:**
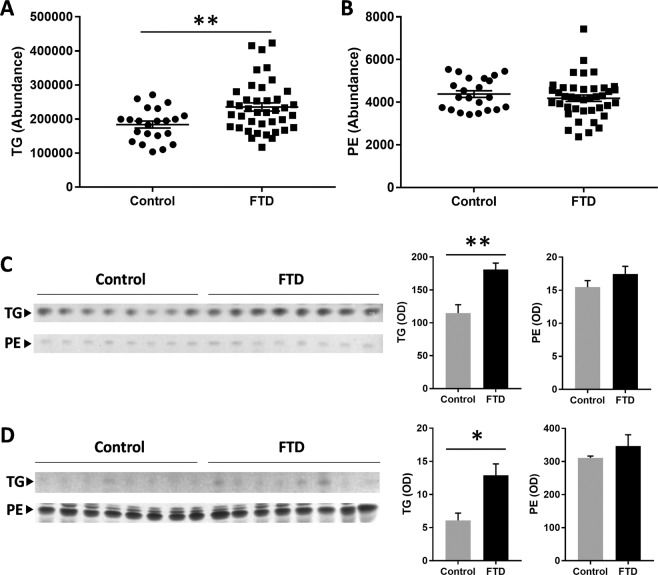
Validation of lipid analysis of FTD serum and brain. (**A**) Triglyceride (TG) was significantly increased in FTD serum (N = 40) compared to controls (N = 22). (**B**) Phosphatidylethanolamine (PE) was unaltered in FTD serum compared to controls. (**C**) Thin layer chromatography (TLC) of serum TG and PE and optical density measurements of the bands. (**D**) TLC of TG and PE in FTD brain (N = 10) compared to control brain (N = 11) and optical density measurements of the bands. Data represent mean and SE as error bars, *P < 0.05, **P < 0.005.

Previous studies based on untargeted lipidomics analysis of FTD blood have revealed global lipid changes in FTD^12^. Here, we used a focused approach to analyze specific serum lipids with the aim of detecting and understanding the pathophysiological changes in FTD. We were interested in three key aspects of FTD pathophysiology that are relevant to neurodegeneration – mitochondrial dysfunction, inflammation, and oxidative stress. We analyzed serum lipids from FTD patients and controls without dementia using sophisticated HPLC-MS and LipidSearch software. A summary of total abundance of all lipids analyzed is shown in Table 1. Firstly, for validation purpose, we compared our new data from the current study to those previously published. We analyzed the levels of two common lipids – triglyceride (TG) and phosphatidylethanolamine (PE) – that are increased and unaltered, respectively, in FTD^11,12^. As expected, TG was significantly increased in FTD serum compared to controls (Fig. 1A), and PE was unaltered (Fig. 1B). To further validate these data, we measured the two lipids in both serum and brain using an alternative method – thin layer chromatography. And once again, TG was significantly increased in both FTD serum and brain (Fig. 1C,D), and PE was unaltered in both FTD serum and brain (Fig. 1C,D).

**Table 1:** A summary of total abundance of serum lipids analyzed.

Lipid	Symbol	Unit	Control	FTD	Change(%)	*P value*
**Lipids**						
Triglyceride	TG	Abundance	183,600 ± 10,190	235,199 ± 11,880	28	0.0017
Phosphatidylethanolamine	PE	Abundance	4,382 ± 156	4,186 ± 151	−4.5	0.3733
Phosphatidylcholine	PC	Abundance	387,876 ± 9,089	362,632 ± 5,998	−6.5	0.0257
Methylphosphatidylcholine	MPC	Abundance	171,355 ± 3,774	151,426 ± 3,305	−11.6	0.0002
**Mitochondrial lipids**						
Cardiolipin	CL	Abundance	12.1 ± 0.5	9.8 ± 0.3	−19	0.0006
Acylcarnitine	AC	Abundance	540 ± 35	381 ± 19	−29	0.0004
**Inflammatory lipids**						
Lysophosphatidylcholine	LPC	Abundance	88,056 ± 5,244	116,117 ± 4,477	32	0.0019
Platelet-activating factor	PAF	Abundance	470 ± 117	1,159 ± 154	147	0.0007
O-acyl-ω-hydroxy fatty acid	OAHFA	Abundance	23 ± 3.3	11 ± 1.4	−52	0.0027
**Lipid aldehydes**						
Acrolein	AL	Abundance	24 ± 2.8	40 ± 5.9	67	0.0279
Malondialdehyde	MDA	nmol/mL	5.1 ± 0.3	5.6 ± 0.4	9.8	0.3630
4-Hydroxynonenal	HNE	μg/mL	0.62 ± 0.08	0.68 ± 0.13	9.7	0.7265

### Detection of mitochondrial dysfunction in FTD using serum lipids

**Figure 2:**
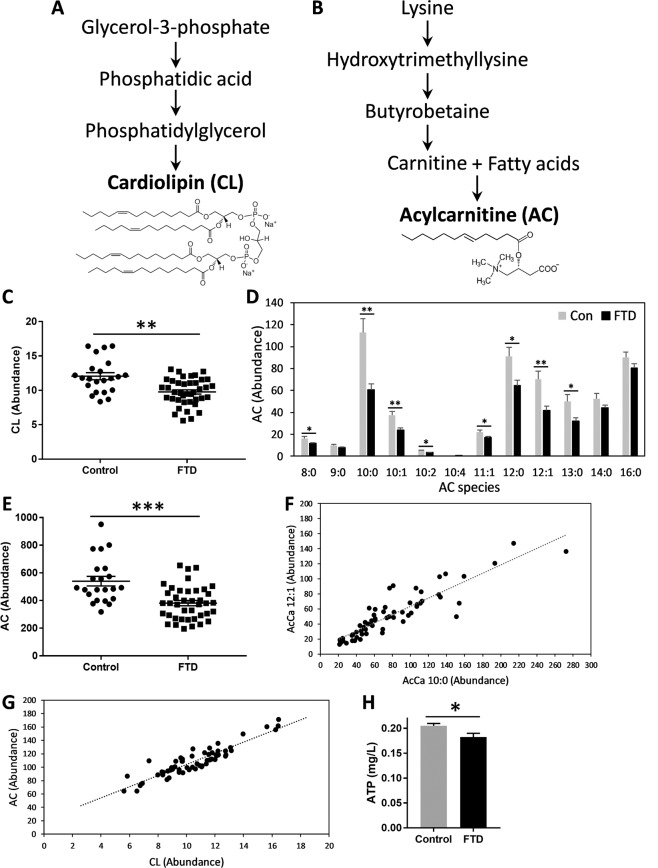
Decreases in mitochondrial lipids and ATP in FTD serum. Biosynthetic pathways of mitochondrial lipids cardiolipin (CL) (**A**) and acylcarnitine (AC) (**B**). (**C**) Total CL levels were decreased in FTD compared to controls. (**D**) Eight of the twelve AC species were decreased in FTD. (**E**) Total AC levels were decreased in FTD. (**F**) A strong correlation between the two most significantly decreased AC species, 10:0 and 12:1 (Pearson’s correlation = 0.897; *P* = 6.4 × 10^−23^). (**G**) An extremely strong correlation between AC and CL levels (Pearson’s correlation = 0.910; *P* = 1.1 × 10^−24^). (**H**) ATP levels were decreased in FTD. FTD (N = 40), controls (N = 22), data represent mean and SE as error bars, *P < 0.05, **P < 0.005, ***P < 0.0005.

Mitochondrial dysfunction is a common pathological feature in Alzheimer’s disease (AD) and Parkinson’s disease (PD)^26^. It is also increasingly evident in FTD^27,28^. Two lipids that play prominent roles in mitochondria are cardiolipin (CL) and acylcarnitine (AC). CL is almost exclusively located in the inner mitochondrial membrane, where it plays roles in numerous enzymatic functions that are involved in mitochondrial energy metabolism^14^. AC acts as a transporter of long-chain fatty acids into the mitochondria, where the fatty acids are oxidized to produce energy, i.e. ATP^15^. CL is a unique phospholipid synthesized from glycerol-3-phosphate (Fig. 2A), whereas AC is a simple lipid consisting of lysine derivative bound to fatty acids (Fig. 2B). The two lipids are structurally different and are synthesized under independent pathways. Changes in CL and AC levels would indicate changes in mitochondrial function/activity.

We compared the levels of CL and AC in FTD and control sera. We identified a single CL species, which was significantly decreased in FTD compared to controls (Fig. 2C). We identified 12 AC species, of which 8 were significantly decreased in FTD (Fig. 2D); the total AC levels were also decreased in FTD (Fig. 2E). The decreases in AC species strongly correlated with one another, for example, the Pearson’s correlation for the two most significantly decreased AC species, 10:0 and 12:1, was 0.897 (*P* = 6.4 × 10^−23^) (Fig. 2F). Importantly, CL levels were strongly correlated to AC levels (Pearson’s correlation = 0.910; *P* = 1.1 × 10^−24^) (Fig. 2G) despite the fact that the two lipids are produced independently. These results strongly suggest mitochondrial dysfunction in FTD. To verify these findings, we then measured ATP level in the same sera. ATP level is a cardinal indicator of mitochondrial function, and it has already been shown that ATP synthesis is decreased in FTD brain and in the brain of mouse models of FTD^27,29^. We found that the ATP levels were significantly decreased in FTD compared to controls (Fig. 2H), supporting our lipid data. When put together, these data support the use of lipid measurements for detecting mitochondrial dysfunction in FTD serum.

### Detection of inflammation in FTD using serum lipids

**Figure 3:**
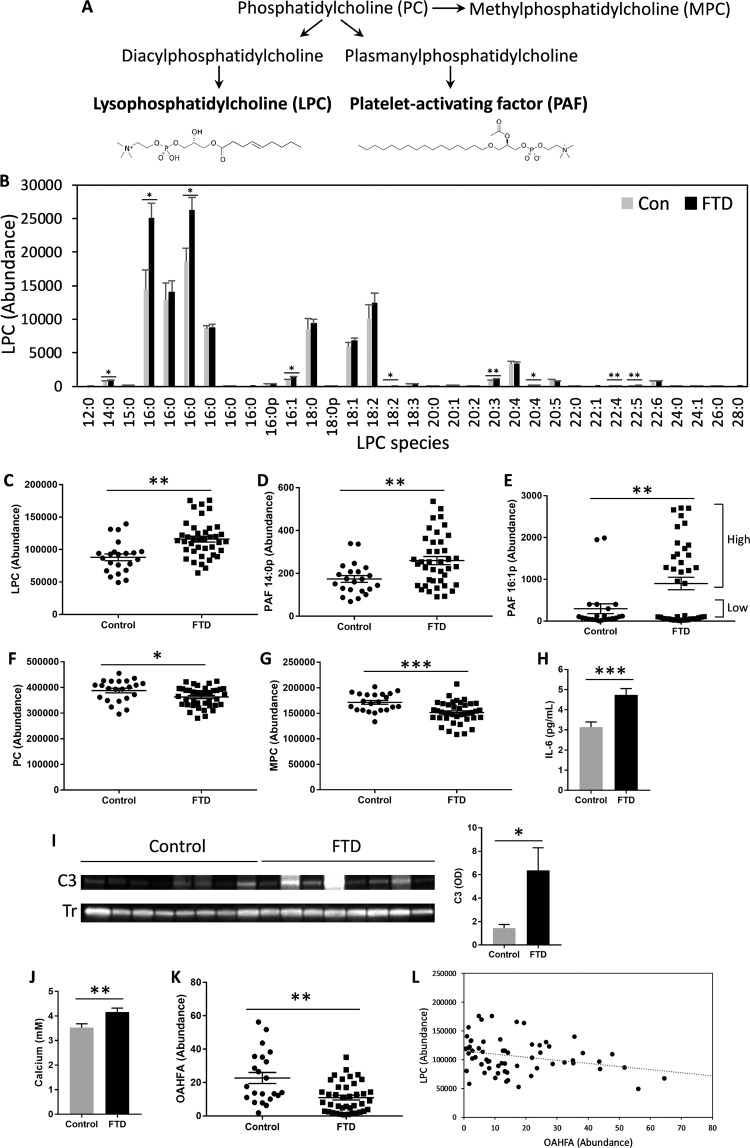
Changes in inflammatory lipids and cytokine in FTD serum. (**A**) Biosynthetic pathways of pro-inflammatory lipids lysophosphatidylcholine (LPC) and platelet-activating factor (PAF), and non-inflammatory lipids, phosphatidylcholine (PC) and methylphosphatidylcholine (MPC). (**B**) The abundance of LPC species, of which 9 were significantly increased in FTD compared to controls. (**C**) The total LPC levels were increased in FTD. (**D**) PAF 14:0p levels were increased in FTD. (**E**) PAF 16:1p levels were increased in FTD. (**F**) The total PC levels were decreased in FTD. (**G**) The total MPC levels were decreased in FTD. (**H**) The pro-inflammatory cytokine IL-6 levels were increased in FTD as measured by ELISA. (**I**) The pro-inflammatory marker C3 levels were increased in FTD as measured by western blotting; normalized by the housekeeper transferrin (Tr) (**J**) Calcium levels were increased in FTD. (**K**) Anti-inflammatory lipid o-acyl-ω-hydroxy fatty acids (OAHFA) levels were decreased in FTD. (**L**) An inverse correlation between LPC and OAHFA (Pearson’s correlation = −0.274; *P* < 0.05). FTD (N = 40), controls (N = 22), data represent mean and SE as error bars, *P < 0.05, **P < 0.005, ***P < 0.0005.

Inflammation is a prominent pathophysiological characteristic in neurodegenerative diseases, including FTD^30,31^. We were interested in whether inflammation in FTD could be detected by analysis of serum lipids. Two serum lipid classes that are known to participate in inflammatory response are lysophosphatidylcholine (LPC) and platelet-activating factor (PAF)^16,17^. LPC and PAF are pro-inflammatory lipids that are also known as second messengers or immediate-response molecules. LPC acts on a number of immune target cells, for example, it upregulates cytokine production and chemotaxis in macrophages and T-lymphocytes^32–35^. It also activates caspase-1 in microglia in the brain neuroinflammatory process^36^. PAF is also a potent activator of inflammatory cells involved in the innate immune system, including neutrophils, macrophages and platelets. It is also a potent neuromodulator^37^. Both LPC and PAF are glycerophospholipids that are derived from the parent lipid phosphatidylcholine (PC) (Fig. 3A). PC and another derivative of PC, methylphosphatidylcholine (MPC) (Fig. 3A), are non-inflammatory lipids.

We compared the levels of LPC and PAF, as well as PC, in FTD and control sera. We identified 33 LPC species, of which 9 were significantly increased in FTD compared to controls (Fig. 3B). The total LPC was also significantly increased in FTD (Fig. 3C). We identified two PAF species (14:0p and 16:1p), both of which were significantly increased in FTD (Fig. 3D,E). Interestingly, there were two distribution clusters for the 16:1p species, i.e. “low” and “high” (Fig. 3E). Most of the control samples (i.e. 91%) were distributed to the low cluster, whereas 50% of the FTD samples were distributed to the low cluster and 50% to the high cluster (Fig. 3E). Within the FTD cohort, the mean of the high cluster was 28.6-fold higher (*P* = 1.1 × 10^−10^) than that of the low cluster with no difference in age (mean age = 65 y for both groups) or gender (male N = 10, female N = 10 for both groups). In terms of correlation, LPC levels were positively correlated to PAF levels (Pearson’s correlation = 0.278; *P* < 0.05). The increase in the lipid levels was specific to only the pro-inflammatory lipids as both non-inflammatory lipids, PC and MPC, were slightly decreased in FTD compared to controls (Fig. 3F,G).

To verify these lipid results, we used two non-lipid assays to measure the level of two independent pro-inflammatory markers in the same sera. Firstly, we used ELISA to measure interleukin 6 (IL-6), which is a major cytokine that regulates inflammatory responses. Polymorphisms of the IL-6 gene are associated with cognitive and behavioral performances of FTD patients^38^. Secondly, we used western blotting to measure complement component 3 (C3), which plays a central role in innate immunity and is commonly used as a blood test for the diagnosis of inflammatory conditions. C3 levels are associated with cognitive decline in FTD patients^39^. We found that both IL-6 and C3 levels were significantly increased in FTD compared to controls (Fig. 3H,I), supporting our lipid data. Furthermore, we analyzed calcium levels as a measure of LPC levels; increases in LPC levels induce increases in calcium levels via G-protein-coupled receptors^40^. Calcium dysregulation is also associated with FTD^41,42^. We found that calcium levels were significantly increased in FTD serum compared to controls (Fig. 3J), once again supporting our lipid data. These findings are consistent with earlier studies that showed pro-inflammatory cytokine levels are increased in FTD brain^43^.

Finally, we analyzed o-acyl-ω-hydroxy fatty acids (OAHFA), which are simple lipids composed of a long carbon-chain fatty acid esterified to an omega-hydroxy fatty acid. They are similar in structure to a group of lipids called fatty acid esters of hydroxy fatty acids (FAHFA). What is interesting is that these lipids exert anti-inflammatory effects^44,45^. We were therefore interested in whether OAHFA levels were altered in FTD. We found that the OAHFA levels were significantly decreased in FTD compared to controls (Fig. 3K), suggesting decreased anti-inflammatory activity. Furthermore, there was a significant inverse correlation between LPC and OAHFA levels (Pearson’s correlation = −0.274; *P* < 0.05) (Fig. 3L). When put together, these data support lipid assays as means to measure inflammation in FTD serum.

### Detection of oxidative stress in FTD using serum lipids

**Figure 4:**
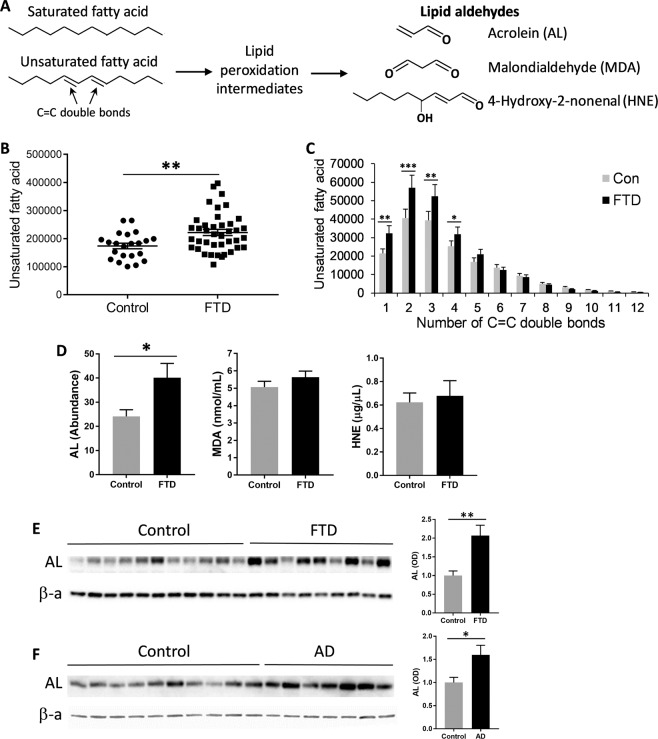
Increases in lipid peroxidation products in FTD serum and brain. (**A**) Unsaturated fatty acids (contain one or more C=C double bonds) are prone to lipid peroxidation, resulting in the formation of lipid aldehydes, acrolein (AL), malondialdehyde (MDA) and 4-hydroxynonenal (HNE). (**B**) The total abundance of unsaturated fatty acid was increased in FTD serum (N = 40) compared to controls (N = 22). (**C**) A comparison of abundance of unsaturated fatty acids with different number of C=C double bonds. (**D**) AL-, MDA- and HNE-conjugated protein levels in FTD serum and controls. (**E**) AL-conjugated protein levels were increased in the superior frontal cortex of FTD brain (N = 10) compared to control brain (N = 11) as detected by western blotting; normalized by the housekeeper β-actin (β-a) (**F**) AL-conjugated protein levels were increased in the superior frontal cortex of AD brain (N = 10) compared to control brain (N = 11). Data represent mean and SE as error bars, *P < 0.05, **P < 0.005, ***P < 0.0005.

In terms of chemistry, saturated fatty acids (i.e. contain no C=C double bonds) are very stable, whereas unsaturated fatty acids (i.e. contain one or more C=C double bonds) are prone to peroxidation (a process of oxidative degradation); the greater the number of C=C double bonds the greater the susceptibility to peroxidation (Fig. 4A). Lipid peroxidation results in the formation of lipid aldehydes – acrolein (AL), malondialdehyde (MDA) and 4-hydroxynonenal (HNE) (Fig. 4A). The three lipid aldehydes are highly reactive, and they conjugate (i.e. bind) to proteins, annulling their function. Increasing evidence indicates that lipid aldehydes contribute to the pathogenesis of neurodegenerative diseases, as well as in normal ageing^46^. It has already been demonstrated that the levels of HNE-conjugated proteins are increased in FTD brain^43,47^. Brain is particularly susceptible to lipid peroxidation due to a high lipid content and a high oxygen consumption.

Firstly, we analyzed the abundance of saturated and unsaturated fatty acids (i.e. in TG) present in the serum; fatty acids are usually derived from TG. The total level of unsaturated fatty acids was significantly increased in FTD compared to controls (Fig. 4B), indicating increased susceptibility to peroxidation (i.e. oxidative stress) in FTD. The significant increases occurred in fatty acids with 1–4 C=C double bonds (Fig. 4C). Secondly, we measured lipid aldehyde-conjugated proteins in the sera. There was a significant increase in the AL-conjugated protein levels in FTD compared to controls, whereas a non-significant trend for increase in the MDA- and HNE-conjugated protein levels (Fig. 4D). To verify these findings, we then measured AL-conjugated protein levels in the superior frontal cortex of FTD (with TDP43 pathology) and control brain, as well as in AD brain (positive control). The AL-conjugated protein levels were significantly increased in FTD and AD compared to controls (Fig. 4E,F), indicating increased oxidative stress in FTD and AD brain. These results support the use of lipid assays to detect oxidative stress in FTD.

## Discussion

Utilization of lipid analysis has helped to understand the pathophysiology of a number of human diseases. With recent advances in mass spectrometry, lipidomics exploration of tissues and fluids has allowed identification of hundreds, if not thousands, of different lipid species, leading to improved diagnosis and development of peripheral biomarkers. In recent years there has been significant interest in the role of lipids in neurodegenerative processes, particularly in the aggregation and propagation of pathogenic proteins, e.g. amyloid-β and α-synuclein. Much of the work in this field has been focused on AD and to a lesser degree PD, with little on FTD. To address this, we asked a pertinent question – what are the changes in serum lipids that indicate FTD pathophysiology. We investigated three key aspects of FTD pathophysiology that are relevant to neurodegeneration, which are mitochondrial dysfunction, inflammation, and oxidative stress. There were two objectives to our investigation – (1) to detect pathophysiological changes in FTD using serum lipids, and (2) identify lipid species and pathways that could be exploited to develop biomarkers for FTD. The overall aim was to provide new insights into an under-recognized perturbed pathology in FTD.

The physiological status of mitochondria can be detected by the analysis of two mitochondrial lipid classes, CL and AC. We measured the levels of CL and AC in the sera, and both were significantly decreased in FTD compared to controls, suggesting mitochondrial dysfunction in FTD. We also verified our lipid data with measurement of ATP levels, which were significantly decreased in FTD serum compared to controls. Our data is consistent with earlier findings that both ATP level and ATP synthase expression/activity are decreased in FTD brain and FTD mouse models^27,29^. ATP synthesis is a major function of mitochondria, and therefore the level of ATP is a sensitive indicator of mitochondrial function/activity. CL and AC are structurally very different and are synthesized under independent pathways, yet their levels correlated with each other extremely strongly, providing further evidence for aberration in mitochondrial function. CL interacts with proteins in the mitochondria in the regulation of a number of mitochondrial processes, including the electron transport chain (i.e. ATP production), cell viability and apoptosis^14,48,49^. It is widely distributed throughout the brain in all cell types, suggesting its importance in the mitochondrial function in the brain. The multi-role of CL is thought to be due to its unique conical-shape structure that renders it to interact with a large array of proteins located within the mitochondrial membranes^50^. In one study, it was shown that CL mediates autophagy of damaged mitochondria in neurons^51^.

AC also plays a number of roles in the mitochondria, e.g. neuroprotection and antioxidant functions^52–54^. Supplementation of AC for the treatment of AD has been trialed. An oral treatment of AC for one year resulted in a slower rate of deterioration compared to placebo-controls, and significantly higher scores in a number of verbal and memory tests^55^. It is unknown whether AC supplementation is of any benefit in FTD. In *in vitro* studies, AC was shown to protect primary neurons and hippocampal cultures against neurotoxic substances^56^. Much work is needed to understand how AC is facilitating neuroprotection in mitochondria.

The second aspect of FTD pathophysiology we investigated using serum lipids was inflammation. For this analysis we targeted two known pro-inflammatory lipid classes, LPC and PAF, both of which are derived from the parent lipid PC. We found that the LPC and PAF levels were significantly increased in FTD compared to controls, whereas the non- inflammatory lipids, PC and MPC, levels were slightly decreased. Increases in LPC and PAF levels would indicate increased inflammatory activity. To verify our lipid data, we measured two key pro-inflammatory markers, IL-6 and C3, and calcium in the same sera and found that all were significantly increased in FTD compared to controls. These data are in accordance with the manifestation of inflammation in FTD^30,31^, in which the levels of IL-6, along with other cytokines, are increased in FTD serum/plasma^57–59^.

LPC regulates cytoskeleton and cellular Ca^2+^ homoeostasis, proliferation, survival, migration and adhesion^40^. PAF is a potent pro-inflammatory mediator implicated in neurodegenerative processes^60^. Initially, it was described as a factor that degranulates (i.e. aggregates) platelets, and thus its name^61^. Its physiological role is diverse and is implicated in a number of diseases^62^. It acts upon numerous inflammation pathways, including enhanced leukocyte adhesion, chemotaxis and leukocyte degranulation^63^. Its production is increased in response to specific stimuli in key host defense cells including macrophages and monocytes. Its level is regulated by PAF acetylhydrolase (PAF-AH), a phospholipase that hydrolyzes the acetyl residue, rendering the lipid inactive^64^. Virtually nothing is known about the role of LPC and PAF in FTD pathophysiology. In AD, increases in PAF levels correlate to the severity of cognitive impairment^65,66^. In one study, it was shown that PAF antagonists enhanced the intracellular degradation of amyloid-β42 in neurons via regulation of cholesterol ester hydrolases^67^. In another study, it was shown that the levels of PAF-AH were higher in AD patients compared to controls^68^.

The presence/absence of C=C double bonds, as well as the number of C=C double bonds, in fatty acids has significant impact on both biophysical and physiological properties of fatty acids. In terms of biophysical properties, unsaturated fatty acids (contain C=C double bonds) have lower melting points and are unstable, whereas saturated fatty acids have higher melting points and are stable. In terms of physiological properties, unsaturated fatty acids are susceptible to peroxidation and its lipid peroxidation products cause oxidative stress or damage to cells and tissues, whereas saturated fatty acids are largely not susceptible to peroxidation. The point of attack by free radicals in the process of lipid peroxidation is the C=C double bonds that contain the methylene -CH2- bridges; the greater the number of double bonds the greater the susceptibility to lipid peroxidation.

Toxic lipid peroxidation products are lipid aldehydes. The three major lipid aldehydes are AL, MDA and HNE; AL is the simplest form of lipid aldehydes (Fig. 4A). They are highly reactive and readily conjugate to proteins, altering or annulling the normal structure and function of proteins. Presence of aldehyde-conjugated proteins indicate cellular oxidative stress and can be used as bioactive markers to detect the extent of lipid peroxidation. In the past, much of the research has been focused on the detrimental effects of lipid peroxidation in the context of atherosclerosis. Accumulation of oxidized LDL in the arteries leads to foam cell formation and plaque development, causing atherosclerosis^69^. Lipid peroxidation is extremely important in the context of brain function since brain is highly enriched in lipids and highly oxygenated, and therefore particularly susceptible to oxidative damage. Growing evidence indicates that increases in lipid aldehydes in the brain are significant contributors to neurodegenerative processes associated with AD. The levels of AL-conjugated proteins are increased in AD brain, cerebral spinal fluid and serum/plasma^70–72^. Likewise, we found that the levels of AL-conjugated proteins were significantly increased in FTD serum and brain, indicating the prevalence of oxidative stress or damage in FTD. In terms of toxicity, AL is particularly toxic as it reacts with glutathione >100 times faster than HNE^73^. When compared to other reactive oxygen species, such as superoxide anion radical, hydrogen peroxide and hydroxyl radical (agents that induce oxidative stress), AL was found to be the most toxic^74–76^.

Lipidomics technology has been increasingly utilized in the analysis of peripheral fluids to understand lipid dysfunction in a number of diseases. Here, we applied lipidomics to detect pathophysiological changes in FTD. We investigated three key aspects of FTD pathophysiology – mitochondrial dysfunction, inflammation, and oxidative stress – that are important to neurodegeneration. Conclusively, all three pathophysiological changes in FTD were detected by the lipid analysis with evidence of similar changes in the brain. This represents the first focused lipidomics analysis of FTD serum that link lipid changes to neurodegeneration. Our study has not only provided useful data in understanding the pathogenesis of FTD, it has also opened avenues for biomarker development and for monitoring disease progression in FTD.
